# Study on Selenium Assimilation and Transformation in Radish Sprouts Cultivated Using Maillard Reaction Products

**DOI:** 10.3390/foods13172761

**Published:** 2024-08-30

**Authors:** Xiaoshuang Zou, Ruiqi Sun, Can Wang, Jun Wang

**Affiliations:** College of Food Science & Nutritional Engineering, China Agricultural University, 17 Qinghua East Road, Beijing 100083, China

**Keywords:** selenoamino acids, reducing sugar, Maillard reaction, radish sprout, HPLC-ESI-MS/MS

## Abstract

The organic selenium (Se), particularly in the form of selenoamino acids, in non-edible sections or by-products of Se-enriched plants, has the potential to generate Maillard reaction products (MRPs) during thermal treatment or fermentation. To elucidate the recycling process of organic selenium in foods and improve the utilization rate of Se, the biotransformation of organic selenium was studied by the cultivation of edible radish sprouts with Se-MPRs. Maillard reactions were simulated using selenoamino acids (SeAAs; selenomethionine and methylselenocysteine) and reducing sugars (glucose and fructose) for preparing Se-MRPs. The structures of the possible dehydrated Se-MRPs were analyzed using a HPLC-ESI-MS/MS system based on their fragmentation patterns and Se isotopic characteristics. Se absorption by the radish sprouts cultivated using Se-MRPs was estimated by the corresponding Se in the SeAAs and the total Se contents. The capabilities of SeAA transformation and total Se assimilation by the sprouts were related to the substrate composition during the Se-Maillard reaction. A particular Se-MRP (selenomethionine + fructose) increased SeAAs transformation by 33.8% compared to selenomethionine. However, glucose and fructose seemed to inhibit the transformation of the Se-MRPs to SeAAs by 10.0 to 59.1% compared to purified Se-MRPs. These results provide key references for the efficient utilization of organic Se in the cultivation of Se-enriched sprouts.

## 1. Introduction

Selenium (Se) is an essential trace element for humans. Se cannot be endogenously synthesized; thus, humans have to obtain it indirectly from the soil through the food chain. Appropriate intake of Se is needed for numerous biological activities in humans, including reproduction, metabolism, the immune system, redox reactions, and anticarcinogenic effects [1,2,3,4]. However, Se deficiency is becoming more prevalent worldwide because of the uneven distribution of this element in the Earth’s crust, leading to approximately 1 billion people living in low-Se belts [5].

Se exists both in inorganic and organic forms. Organic Se exhibits higher bioaccessibility and bioavailability than inorganic Se [6]. Generally, Se-containing compounds are produced by plants through biotransformation and are available as organic Se (mostly selenomethionine (SeMet) and methylselenocysteine (MeSeCys)). Thus, Se-enriched foods are suitable as dietary supplements for the Se-deficient population. Se-enriched agricultural products can be obtained from Se-enriched areas. However, their production patterns are constrained by geographic factors and transportation. Fortunately, agronomic Se biofortification is a feasible way to obtain Se-enriched agricultural products and has successfully mitigated the low Se levels in food [7,8]. Se-enriched vegetables have a higher Se content and are regarded as the primary source of Se in the diet [9,10]. Among Se-accumulating vegetables, sprouts are an excellent dietary source and are commonly consumed in Asia, where Se deficiency is prevalent [11]. Sprouts also show advantages, including a short growing period, feasibility of hydroponic cultivation, and lack of seasonal restrictions [12,13]. However, the bioavailability of Se to plants mainly depends on the chemical forms of Se. Selenate and selenite are typically used in Se biofortification via the sulfur assimilatory pathway. A higher Se uptake can be achieved using organic Se [14]. Organic Se is environmentally friendly and can be obtained from the inedible parts of Se-enriched plants or by-products, which are cost-effective and abundant resources. These organic Se-containing materials can be used as exogenous Se for cultivating Se-enriched crops. However, a certain amount of reducing sugar (mainly glucose and fructose) is present in these raw materials. Thermal treatment and fermentation inevitably occur during the storage, extraction, and processing of these materials. Hence, a Maillard reaction would happen to SeAAs like common amino acids [15]. There have been few studies on the bioaccessibility of Se-Maillard reaction products (Se-MRPs) to crops. Moreover, the presence of reducing sugar is an undetermined factor for the absorption of Se compounds by plants. Filling these knowledge gaps would be of great interest to the advancement of Se recycling and the usage in Se-enriched foods production.

As SeMet and MeSeCys are common species found in Se-enriched plants, here, the glycation reaction of SeMet and MeSeCys with reducing sugars was used to prepare Se-MRPs. In the present study, the uptake of Se-MRPs was investigated for the first time using radish sprouts, a common edible Se-accumulating vegetable. This paper aimed to provide a scientific explanation for the production of Se-enriched vegetables with an alternative type of exogenous organic Se from Se-MRPs.

## 2. Materials and Methods

### 2.1. Chemicals and Materials

Radish seeds (JingRed No. 4_F1_) were purchased from Jingyan Yinong Seed Sci-Tech Co., Ltd. (Beijing, China). Analytical standard-grade selenocystine (SeCys_2_), SeMet, MeSeCys, iodoacetamide, dithiothreitol (DTT), HPLC-grade methanol (MeOH), ethanol, glycerol, glucose (Glu), fructose (Fru), sodium carbonate (99.5%), Dowex AG 50W-X8 resin, potassium ferricyanide (99.5%), and trifluoroacetic acid (TFA) were purchased from J&K Scientific Ltd. (Beijing, China). Protease XIV (3.5 U/mg) was procured from Sigma-Aldrich (St. Louis, MO, USA). Ultrapure water was produced using a Synergy UV system (Millipore, Bedford, MA, USA).

### 2.2. Synthesis of Se-MRPs

The Se-Maillard reaction system was simulated based on the work of Wang et al. [16], with modifications. Briefly, 5 mg of SeAAs was mixed with 10 mg of Glu or Fru in 5 mL of aqueous solutions of 70%, 80%, and 90% glycerin. The pH of these solutions was adjusted to 9.0 with Na_2_CO_3_. Then, refluxing was performed at 110, 120, and 130 °C, respectively, for hours. Finally, the reaction solution was adjusted to 10 mL with ultrapure water. The progress of the Se-Maillard reaction was monitored by the content of unreacted SeAAs using a HPLC-ESI-MS/MS system. A volume of 0.1 mL was sampled and adjusted to 10 mL for each measurement.

### 2.3. Purification of Se-MRPs

An ASE-12 solid-phase extraction (SPE) apparatus (Tianjin Aotesaisi Instrument Co., Ltd., Tianjin, China) was used for the purification of Se-MRPs, based on the method of Yuan et al. [17], with modifications. The SPE column was packed with Dowex AG 50W-X8 resin. Approximately 1 mL of Se-MRP solution was loaded onto the column. Then, the column was washed with 20 mL of ultrapure water to remove glycerol and unreacted reducing sugar. The samples were eluted using 80 mL of a 0.5 M ammonia solution. The filtrate was completely dried using a rotary evaporator at 55 °C. Finally, the samples were redissolved with ultrapure water and adjusted to the initial volume (1 mL) for further analysis.

### 2.4. Sprout Germination and Cultivation

The germination and cultivation of sprouts were carried out light-free according to our previous work [13]. Radish seeds were shielded from light and soaked in ultrapure water for germination (12–16 h) before placing them into a CB-321 bean sprout container (GONNIE, Foshan, China) at 25 °C. Radish sprouts were first grown in 500 mL of ultrapure water for 24 h in four cycles. Based on the total Se measured by atomic fluorescence spectroscopy (AFS), the total Se content of all Se-containing solutions was adjusted to 0.041 mg/L. Then, the samples were exposed to 500 mL of Se-containing solutions for 24 h. Finally, the sprouts were again grown in ultrapure water for another 24 h. SeMet and MeSeCys were set as blank groups. Untreated SeMet-Fru and MeSeCys-Fru were set as controls. The total cultivation period was set as 144 h. All samples were cleaned with ultrapure water before further processing.

### 2.5. Extraction of Organic Selenoamino Acids

Ultrasonication and digestion were performed to thoroughly release SeAAs in free and protein-bound states. Approximately 5.0 g of radish sprouts were homogenized with 40 mL of ultrapure water before the ultrasonic extraction was carried out in a constant temperature bath at 25 °C for 30 min. Then, the samples were digested and placed in a water bath (SHA-CA, Changzhou Putian Instrument Manufacturing Co., Ltd., Changzhou, China) with 0.25 mg/mL of protease XIV at 47 °C for 2 h. Finally, the solutions were centrifuged (TLG-16M, Hunan Xiangyi Centrifuge Instrument Co., Ltd., Changsha, China) at 4000 rpm for 15 min, and the supernatant was filtered through a 0.22 μm polyethersulfone (PES) filter. The filtered supernatant was used for the detection of both free and combinative organic SeAAs.

### 2.6. Sample Analysis

#### 2.6.1. Preparation of Calibration Curves

Standard stock solutions of SeAAs were prepared in ultrapure water with concentrations of 102 mg/L (CAM-SeCys), 104 mg/L (MeSeCys), and 100 mg/L (SeMet). All solutions were stored at 4 °C. Quantification was carried out using an external calibration method. Calibration standards were diluted to concentrations of 5, 10, 20, 40, 100, and 200 μg/L in the hydrolysate matrices.

#### 2.6.2. Analysis of Organic SeAAs

Based on our previous work [18], qualitative and quantitative analyses of SeAAs in radish sprout samples were performed on an Agilent 1260 liquid chromatograph coupled with a 6460 electrospray ionization triple quadrupole mass spectrometer. DTT and iodoacetamide were used in the derivatization of SeCys_2_ and SeCysH into CAM-SeCys for better determination. The total Se content was determined based on the atomic fluorescence spectrum (AFS-9230, Beijing Jitian Instrument Co., Ltd., Beijing, China).

#### 2.6.3. Analysis of Se-MRPs

Se-MRPs were detected according to the method of Yuan et al. [19], with modifications. Detection parameters were as follows: injection volume of 10 μL, column temperature of 30 °C, and flow rate of 0.15 mL/min. The mobile phases were ultrapure water (phase A) and acetonitrile (phase B). The isocratic elution program was performed with a mobile phase ratio of 70% B. The operating conditions for mass spectrometry were as follows: sheath gas temperature of 350 °C, gas flow of 8 L/min, nebulizer pressure of 45 psi, and capillary voltage of 3800 V. Product ion^+^ mode was used for sample analysis.

### 2.7. Statistical Analysis

All measurements were performed at least in triplicate, and the results were expressed as the mean ± SD. One-way ANOVA with multiple comparisons employing the LSD test was used to compare the means among different treatments (*p* < 0.05). The data were analyzed using Statistix 8.0 (Analytical Software, Tallahassee, FL, USA). The graphs were plotted using Origin 2017 (Origin Lab, Northampton, MA, USA).

## 3. Results and Discussions

### 3.1. Preparation of Se-MRPs

The structures of SeAAs are similar to those of their corresponding sulfur-containing amino acids. Maillard reactions inevitably occur between the SeAAs and reducing sugars during the extraction and fermentation of organic Se-containing materials [20]. The carbonyl group of reducing sugars occurs with the amino group of selenoamino acids to generate the condensation product N-substituted glycosilamine, which rearranges to form the corresponding primary reaction product of Maillard [21]. Thus, two common SeAAs and reducing sugars were used to prepare Se-MRPs for evaluating the assimilation and transformation of Se by plants. Alkaline heating conditions were favorable to increase the reaction rate and variety of products for better simulation of the Se-Maillard reaction [22]. HPLC-ESI-MS/MS was used to detect the content of unreacted SeAAs to monitor the reaction progress indirectly.

SeAAs seemed to barely react with reducing sugars in ultrapure water, even under alkaline heating conditions. Because a high moisture content is not conducive to the occurrence of the Se-Maillard reaction, glycerol (a commonly used solvent in the Maillard reaction) was added to the solution. With the addition of glycerol, the water activity decreased, and the boiling temperature of the reaction system gradually increased. The addition of glycerol appeared to facilitate the Se-Maillard reaction much more readily in an aqueous solution under alkaline conditions (pH = 9.0). Decreasing the water activity and increasing the refluxing temperature accelerated the consumption of SeAAs for the formation of Se-MRPs [23].

**Figure 1:**
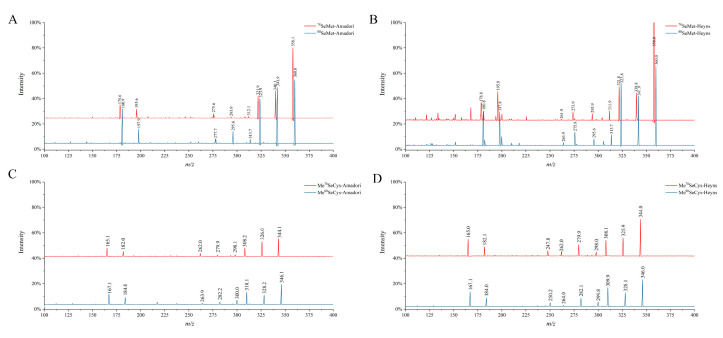
Mass spectra of Se-Amadori and Se-Heyns by product ion scan mode. (**A**): Mass spectra of ^78^SeMet-Amadori and ^80^SeMet-Amadori, (**B**): Mass spectra of ^78^SeMet-Heyns and ^80^SeMet-Heyns, (**C**): Mass spectra of Me^78^SeCys-Amadori and Me^80^SeCys-Amadori, (**D**): Mass spectra of Me^78^SeCys-Heyns and Me^80^SeCys-Heyns.

To further confirm that SeAAs could react with reducing sugar via the Maillard reaction, the structures of Se-Amadori and Se-Heyns compounds were analyzed by the fragmentation patterns of their corresponding compounds in the early stages of the reaction [16,19]. As shown in Figure 1, the ^78^Se and ^80^Se fragment ions of SeMet-MRPs and MeSeCys-MRPs were compared with the mass spectra obtained in the product ion scan mode. Based on the characteristics of the Se isotopes and the cleavage rules, it can be deduced that the Se-Maillard reaction occurred and the primary products (Se-Amadori or Se-Heyns) were generated during the reaction.

**Figure 2:**
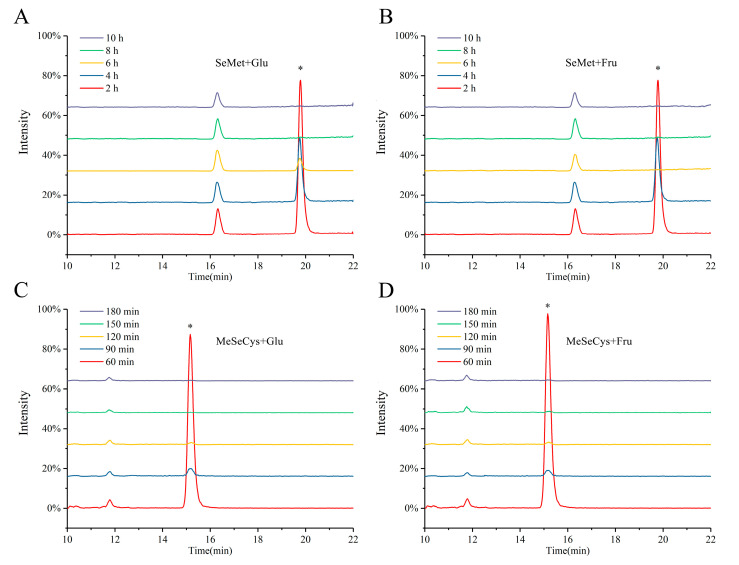
Mass spectra of unreacted selenoamino acids contents in the preparation of different Se-MRPs by MRM mode. (* indicated the residual contents of Selenamino acids.). (**A**): MRM spectra of SeMet during the preparation of SeMet-Glu-MPRs, (**B**): MRM spectra of SeMet during the preparation of SeMet-Fru-MPRs, (**C**): MRM spectra of MeSeCys during the preparation of MeSeCys-Glu-MPRs, (**D**): MRM spectra of MeSeCys during the preparation of MeSeCys-Fru-MPRs.

High temperature contributed to accelerate the reaction progress. However, excessive temperature resulted in an uncontrollable reaction process. The reflux at 120 °C in a 90% aqueous glycerol solution was selected with a faster reaction rate and more abundant reaction products. Data showed that the unreacted SeAA contents of all groups decreased as the reaction time increased (Figure 2). Based on the time needed to reach the reaction endpoints (i.e., when all the SeAAs were consumed), the reaction duration was set at 8 h for SeMet-Glu-MRPs, 6 h for SeMet-Fru-MRPs, and 120 min for both MeSeCys-Glu-MRPs and MeSeCys-Fru-MRPs. The observed higher consumption rate of MeSeCys than that of SeMet indicated that the progress of the Se-Maillard reaction relied on the composition of substrates crucially. Thus, performing a structural analysis of Se-MRPs is essential for gaining deeper insights into the reaction conditions required for the produced Se-containing compounds.

### 3.2. Molecular Recognition of Se-MRPs

**Figure 3:**
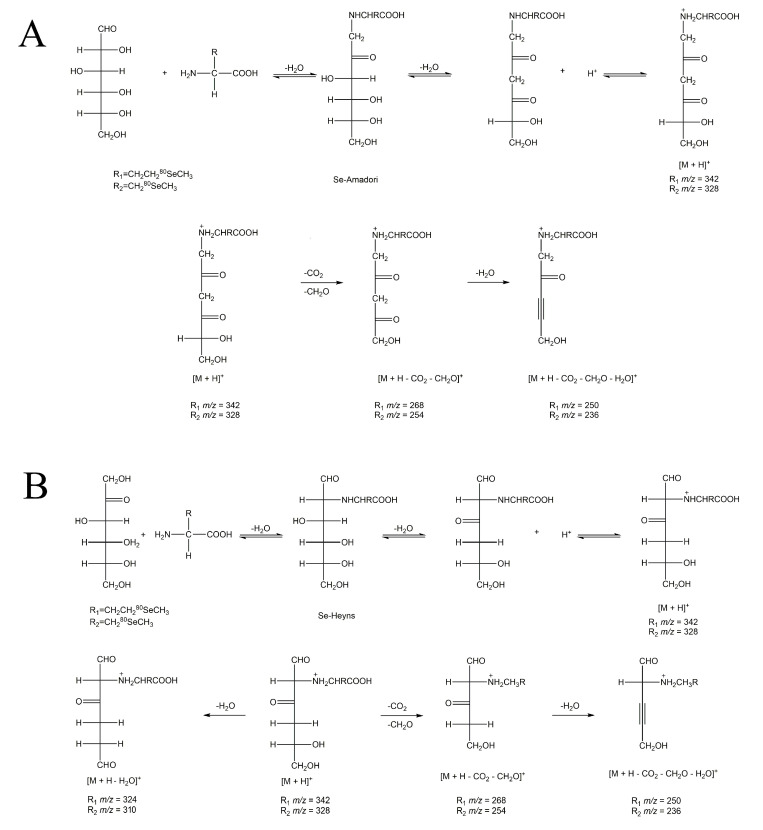
Proposed fragmentation pathways of Se-Amadori (**A**) and Se-Heyns (**B**) cleavage products.

The primary MRPs would further dehydrate, rearrange, and decompose to produce smaller molecules under prolonged reaction duration conditions. The occurrences of the Se-Maillard reaction were confirmed by qualitative analysis of Se-Amadori and Se-Heyns in the early stage of the reaction. However, neither of the primary Se-MRPs was detected at the reaction endpoints of either substrate combination, indicating that pyrolysis occurred. Therefore, the product ion scan mode of mass spectrometry was used to analyze the structures of the compounds generated from Se-Amadori and Se-Heyns and their cleavage products at the reaction endpoints. As shown in Table 1, dehydration products with mass-to-charge ratios (*m/z*) of ^80^Se 342 (^78^Se 340) for SeMet-MRPs and ^80^Se 328 (^78^Se 326) for MeSeCys-MRPs were qualitative analysis based on the properties of the Se isotopes. According to the data of product ions, similar fragmentation patterns were obtained to confirm these Se-containing dehydration products, and the formation and possible fragmentation patterns of Se-MRPs are illustrated in Figure 3. The molecular recognition of Se-MRPs may contribute to our understanding of Se assimilation and transformation by plants.

### 3.3. Assimilation and Transformation of Se-MRPs by Radish Sprouts

Both inorganic and organic forms of Se can be applied for the cultivation of Se-enriched plants. Our previous work demonstrated that SeAAs were absorbed and transformed into free MeSeCys by sprouts when the fortified SeAAs concentration was 0.10 mg/L [13], and the results showed that the Se in SeAAs represented at least 90% of the total Se content in the sprouts cultivated using SeAAs. The remaining Se might exist in other Se-containing compounds rather than SeAAs. Thus, the Se content in SeAAs could be regarded as the primary indicator of the Se assimilation. Nevertheless, this has not been corroborated in radish sprouts cultivated using other Se-containing compounds. In this study, the prepared Se-MRPs were used for the cultivation of Se-enriched radish sprouts. The Se content of SeAAs in relation to the total Se content indicates that SeAAs does not represent the total organic Se in sprouts cultivated with reactants or Se-MRPs. Moreover, the generation of volatile Se due to the cleavage and rearrangement of SeAAs under alkaline heating conditions resulted in Se losses. This agreed with the study of Khanam & Platel [24], which reported that substantial decreases were observed in the Se contents of heated Se-enriched foods. In order to ensure a consistent total Se content for each group, total Se at 0.041 mg/L was applied. This concentration was equivalent to that of Se in 0.10 mg/L of SeAAs (SeMet). In the analysis of organic Se, enzymatic hydrolysis was performed to guarantee that potentially combinative SeAAs could be released from the sprouts cultivated with Se-MRPs. In this study, radish sprouts cultivated utilizing Se-MPRs featuring a total Se concentration of 0.041 mg/L exhibited a total Se content ranging between 0.039 and 0.178 mg/kg FW. The results were similar with the total Se content of the radish sprouts cultivated using the Na_2_SeO_3_ solution seed soaking method with a similar total Se concentration after calculation. Upon calculation, the total Se content (DW) of the results was found to be comparable to that of sprouts cultivated by the Na_2_SeO_3_ solution seed soaking method utilizing similar total Se concentrations [25]. These underscored the efficacy of Se-MPRs in fostering the growth of Se-enriched radish sprouts.

#### 3.3.1. Total Se Content

The total Se contents in the sprouts represent a more objective and intuitive way to evaluate the Se assimilation capability of sprouts grown in different Se-containing culture solutions. To determine the total Se assimilation capability of radish sprouts toward the reactants and Se-MRPs, the influence of additives (Na_2_CO_3_, glycerol, and reducing sugars) in the culture solutions needs to be evaluated before cultivation using Se-MRPs. Therefore, the total Se content was detected by applying the AFS method. The results showed that there were no significant differences (*p* > 0.05) in the total Se contents of the sprouts compared to the SeAAs control groups, regardless of the presence of Na_2_CO_3_ or glycerol. These results demonstrated that Na_2_CO_3_ and glycerol did not have any effects on the total Se assimilation by the radish sprouts.

**Figure 4:**
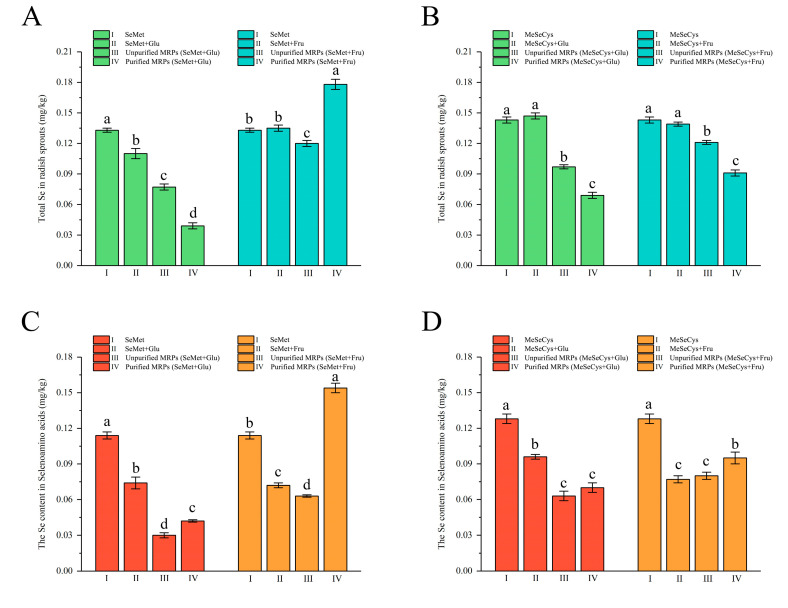
Total Se assimilation and SeAAs transformation capabilities of sprouts to different reactants and Se-MRPs (mean ± SD, *n* = 3). Different letters (a, b, c, and d) indicate statistical differences at *p* < 0.05). (**A**): The total Se contents of cultivated by SeMet systems, (**B**): The total Se contents of cultivated by SeMeCys systems, (**C**): The SeAAs contents of cultivated by SeMet systems, (**D**): The SeAAs contents of cultivated by SeMeCys systems.

The data on the cultivated samples are shown in Figure 4A,B. A significant Se decrease in the SeMet+Glu group was observed (*p* < 0.05). The total Se contents slightly changed in the SeMet+Fru, MeSeCys+Glu, and MeSeCys+Fru groups, with no significance (*p* > 0.05). This indicates that only glucose had a negative effect on the total Se assimilation capability for sprouts cultivated with SeMet. However, for the unpurified Se-MRP groups, the total Se contents of all the cultivated sprouts were significantly lower than those of the SeAAs control groups (*p* < 0.05). This may be due to the formation of Se-MRPs, which affected the total Se assimilation capability. A lower total Se was observed in the SeMet+Glu, MeSeCys+Glu, and SeMet+Fru groups as a result of the removal of reducing sugars and other interfering substances. This proved that the main components of these Se-MRPs were not conducive to the assimilation of total Se. Nevertheless, the purified MRPs (SeMet+Fru) group exhibited excellent Se assimilation capability with a total Se content that was 33.8% higher than that of the SeAA control group. Therefore, we can infer that fructose inhibited the total Se assimilation capability of the MRPs (SeMet+Fru) by the sprouts. The purified MRP (SeMet+Fru) was thus feasible for the cultivation of Se-enriched radish sprouts.

#### 3.3.2. SeAAs Content

To further investigate the Se transformation capability of Se-MRPs, we used HPLC-ESI-MS/MS to analyze the composition of SeAAs in the sprouts. Based on the isotopic properties of Se, which were verified in our previous work [11], we simultaneously monitored SeAAs structured by ^80^Se and ^78^Se, using the multiple reaction monitoring (MRM) mode. The ratio of the peak area (2.09) of ^80^Se to ^78^Se and the retention times are feasible indicators for the qualitative analysis. The data showed that only the presence of free MeSeCys could be determined by the peak area ratio and retention time in sprouts cultivated with Se-MRPs. In the SeAA control groups, SeAAs represented over 90% of the total Se contents. Furthermore, the effects of additives on the SeAA transformation capability were also determined. Na_2_CO_3_ and glycerol had no significant (*p* > 0.05) effects on the SeAAs transformation capability compared to the SeAA control groups. However, the addition of glucose and fructose significantly (*p* < 0.05) lowered the free MeSeCys contents of the groups cultivated with selenoamino acids compared to the control groups. Therefore, it can be deduced that the reducing sugars inhibited the capability of transforming SeAAs in the solution to SeAAs in the sprouts. Glucose simultaneously suppressed both Se assimilation and the SeAAs transformation capability of SeMet.

The SeAA contents of sprouts cultivated with Se-containing solutions are displayed in Figure 4C,D. There were nonnegligible differences between SeAAs and the total Se contents because of the reducing sugars. For the SeMet+Glu group, the presence of glucose decreased the total Se content by 17.3% and decreased the SeAAs content by 40.6%. There were also decreases in the SeAA contents compared to the total Se of the corresponding SeAAs control groups: 45.9% for SeMet+Fru, 32.9% for MeSeCys+Glu, and 46.2% for MeSeCys+Fru. The hindered SeAAs transformation and unchanged total Se assimilation capability indicate that other unknown Se-containing compounds were converted as a result of the addition of reducing sugar. Furthermore, lower SeAA contents were detected in both unpurified MRP (SeMet and MeSeCys) groups. The trends of the SeAA contents differed from those of the total Se contents after the purification with Dowex resins. The SeAA contents represented 38.5% to 66.1% of the total Se contents. These differences indicated either inhibition of MeSeCys transformation or that other Se-containing compounds were produced due to the formation of MRPs. As the reported by Zagrodzki et al., when selenoesters of six kinds were used to cultivate kale sprouts, these Se-containing compounds underwent not only conversion into selenoamino acids but also transformation into various other Se-containing forms [26].

Significantly increased SeAA contents (*p* < 0.05) demonstrated the negative effects of reducing sugars on Se transformation. The SeAA contents of purified MRPs represented 86.5–107.6% of the total Se content. This indicated that the primary organic form of Se was MeSeCys in sprouts cultivated with purified MRPs. However, only the group of purified MRPs (SeMet+Fru) showed an increase in the SeAA content compared to the SeAA control group (*p* > 0.05). These results suggest that the purified MRPs (SeMet+Fru) enhanced the SeAAs transformation capability. Furthermore, sprouts cultivated with the purified MRP (SeMet+Fru) also showed a 33.8% higher total Se content. These results indicate that the purified MRP (SeMet+Fru) was a more favorable Se fortifier than the other Se-MRPs.

In summary, higher Se assimilation and SeAAs transformation capability were achieved by controlling the composition of the reaction substrates, as well as the reaction conditions. The research data in this study provide important references for the further investigation of Se assimilation and transformation in radish sprouts cultivated using organic Se compounds.

## 4. Conclusions

In this study, two common SeAAs and reducing sugars were used to simulate the formation of Se-MRPs during the extraction and processing of Se-containing materials. The corresponding dehydrated Se-Amadori and Se-Heyns compounds of SeMet and MeSeCys were molecularly recognized by their fragmentation patterns and Se isotope properties, using HPLC-ESI-MS/MS in product ion scan mode. The radish sprouts were cultivated with the reactants and Se-MRP culture solutions at the same total Se concentration. The SeAA transformations in the radish sprouts were suppressed because of the presence of reducing sugars in all groups. Glucose simultaneously inhibited both total Se assimilation and SeAA transformation in the SeMet+Glu group. However, no significant change in the total Se content was observed for the groups cultivated with SeMet+Fru, MeSeCys+Glu, and MeSeCys+Fru. Furthermore, reducing sugars had a negative effect on the absorption of Se-MRPs, as the removal of these sugars by purification led to increases in the contents of SeAAs in all cultivated groups. Only the purified MRP (SeMet+Fru) exhibited excellent Se absorption in radish sprouts in contrast to other groups, both in terms of SeAAs transformation and total Se assimilation. Thus, the reaction substrates are responsible for Se absorption from the Se-MRPs. This study provides scientific evidence for adjusting the reaction conditions of Se-MRPs used in the soilless cultivation of Se-enriched sprouts.

## Figures and Tables

**Table 1:** Precursor ions and product ions of Se-MRPs.

Analytes	Precursor Ion (*m/z*)	Product Ion (*m/z*)
MRPs (^80^SeMet+Glu)	342	268, 250
MRPs (^78^SeMet+Glu)	340	266, 248
MRPs (^80^SeMet+Fru)	342	324, 268, 250
MRPs (^78^SeMet+Fru)	340	322, 266, 248
MRPs (Me^80^SeCys+Glu)	328	254, 236
MRPs (Me^78^SeCys+Glu)	326	254, 234
MRPs (Me^80^SeCys+Fru)	328	310, 254, 236
MRPs (Me^78^SeCys+Fru)	326	308, 254, 234

## Data Availability

The original contributions presented in the study are included in the article; further inquiries can be directed to the corresponding author.
